# Effects of Mobile Health Care App "Asmile" on Physical Activity of 80,689 Users in Osaka Prefecture, Japan: Longitudinal Observational Study

**DOI:** 10.2196/65943

**Published:** 2025-05-21

**Authors:** Asuka Oyama, Kenshiro Taguchi, Hiroe Seto, Reiko Kanaya, Jun'ichi Kotoku, Miyae Yamakawa, Hiroshi Toki, Ryohei Yamamoto

**Affiliations:** 1 Health and Counseling Center The University of Osaka Toyonaka Japan; 2 Graduate Degree Program of Health Data Science Teikyo University Tokyo Japan; 3 Graduate School of Human Sciences The University of Osaka Suita Japan; 4 Division of Health Sciences Graduate School of Medicine The University of Osaka Suita Japan; 5 Graduate School of Medical Care and Technology Teikyo University Tokyo Japan; 6 Department of Nephrology Graduate School of Medicine The University of Osaka Suita Japan; 7 Laboratory of Behavioral Health Promotion, Department of Health Promotion Graduate School of Medicine The University of Osaka Suita Japan; 8 Institute for Sports and Global Health The University of Osaka Suita Japan

**Keywords:** physical activity, step counts, mobile health, mHealth, app, Causal Impact, Causal Inference, smartphone, mobile phone, exercise, apps, observational study, Osaka, Japan, healthy lifestyle, lifestyle-related disease

## Abstract

**Background:**

Lifestyle-related diseases can be controlled by improving individuals’ lifestyles; however, improving and maintaining a healthy lifestyle is difficult. Mobile health (mHealth) applications have recently attracted attention as tools for maintaining and improving health, and their use may also increase physical activity.

**Objective:**

This study aimed to verify the effect of registration in Asmile, an mHealth application provided by the Osaka Prefectural Government, on step counts using a Causal Impact approach based on the step count data recorded in the Asmile application.

**Methods:**

This observational study included Osaka residents aged 20-79 years, newly registered to Asmile, between the fiscal years 2020 and 2023. Of these, 80,689 participants with step count records for 4 weeks before and after the day they registered to Asmile were included in the analysis. We used daily step counts that were automatically transferred from a standard smartphone health care app into Asmile. We used a Causal Impact model to estimate the increase in step count after registration to Asmile.

**Results:**

Of the 80,689 participants analyzed, 38.5% (31,082/80,689) were men, and the mean age was 51.6 (SD 13.2) years. The mean step count before registration was 5923 (SD 4860) steps per day, with the highest proportion of new users registered in spring (38,389/80,689, 47.6%) and in fiscal year 2020 (34,491/80,689, 42.7%). The analysis revealed that the effect of Asmile registration on step counts was 360 steps (95% CI 331-389) per day and 10,041 steps (95% CI 9632-10,450) over 4 weeks. Stratified analysis showed that the impact of increased step count was more pronounced in younger groups and groups with fewer step counts before registration. Conversely, the effect of registration on step count was relatively minor in the groups registered in summer or winter.

**Conclusions:**

This study demonstrates increased physical activity among users registered with the Asmile app. These findings suggest that mHealth apps such as Asmile can effectively promote healthier lifestyles and potentially reduce the risk of lifestyle-related diseases.

## Introduction

Lifestyle-related diseases, such as cancer, heart disease, stroke, diabetes, and hypertension, are influenced by daily eating and exercise habits, which contribute to their onset and progression [1-6]. Maintaining a healthy lifestyle is essential for preventing these diseases. Several studies have reported that increasing step counts and physical activity improve mortality risk, cardiovascular disease, and dementia [7,8]. However, improving and maintaining a healthy lifestyle is difficult.

Mobile health (mHealth), a health care support system that uses mobile devices to provide health care services, has recently attracted attention as a tool to maintain and improve health. Several studies have shown that mHealth apps contribute to weight loss [9], blood pressure reduction [10], cardiovascular disease (CVD) risk reduction [11], and cognitive function improvement [12,13]. Thus, mHealth apps are beginning to be recognized as valuable tools for various purposes in health promotion.

However, previous studies have yet to reach a consensus regarding the improvement of exercise habits through the use of mHealth apps. Yerrakalva et al [14] conducted a meta-analysis to investigate the effects of mHealth app interventions on physical activity in older adults aged 55 years and older. Although the mHealth intervention showed a trend toward increased step counts, no statistically significant results were found. Similarly, Direito et al [15] conducted a meta-analysis on the effect of mHealth apps on physical activity in free-living young adults but found no statistically significant effect. By contrast, Hamaya et al [16] examined the impact of an increase in step counts taken by 12,602 app users in the years before and after using the mHealth app and found an increase of 510 steps per day. However, this finding was only a simple before and after comparison, and the effects of multiple events that promoted an increase in step count after registration were also included. Therefore, this result was insufficient to support the hypothesis that using the mHealth app increases physical activity.

Moreover, there are limitations to the methods that are widely used to evaluate the effectiveness of mHealth apps. Difference-in-differences (DID) [17-19] is a standard approach to estimating pre- and postintervention causal effects. It can estimate causality between the pre-and postintervention differences in the intervention group and the control group. However, DID is often unavailable for analyses using the mHealth app because it is impossible to set a control group. In addition, DID analysis frequently considers only 2 time points: preintervention and postintervention. Thus, it is not well suited to capture how intervention effects evolve. Therefore, epidemiological studies on the increase in step count in large populations using an estimation model of causal effects that replace DID are essential for statistically and quantitatively evaluating the impact of mHealth apps on physical activity.

This observational study involved 80,689 new registrants of the Asmile health care app developed by the Osaka Prefecture. It aimed to verify the short-term effect of mHealth registration on the increase in step counts using a causal inference model called “Causal Impact,” based on the step count data recorded in the Asmile app. To the best of our knowledge, this is the first large population observational study quantifying the effect of mHealth on physical activity using a time series causal estimation model.

## Methods

### mHealth Care App “Asmile”

Released in 2019, Asmile is a mobile health care app the Osaka Prefectural Government provides on iOS and Android [20]. This app aims to improve residents’ lifestyle habits and prevent lifestyle-related diseases. It is available free of charge to residents of Osaka Prefecture aged 18 years or older. As of June 2024, there are more than 400,000 Asmile users. Since this health app is available only to residents of Osaka Prefecture, it is difficult to compare its popularity and awareness with health apps available nationwide. However, with approximately 50,000 daily active users, Asmile is one of the most popular health apps in Osaka. One of the distinctive features of Asmile is its automatic integration with the standard functions of smartphones, enabling users to track their daily steps within the app seamlessly. In addition, users can manually record other daily lifestyle habits (weight, body temperature, sleeping hours, breakfast intake, tooth brushing, etc) using the Asmile app.

The Asmile app awards points for daily healthy behaviors such as achieving step count goals, eating breakfast, recording tooth brushing, recording weight, answering in-app health questionnaires conducted within the app, and reading near-daily health columns. The points can be used to participate in weekly and monthly lotteries, which offer rewards such as electronic money. This feature is unique to the Asmile app, and by providing daily incentives for achieving target step counts, the app may encourage users to increase their daily step counts. Previous studies have also suggested that using a digital incentive system for the step following could boost step counts [21,22]. In addition, the Asmile app provides daily health columns, which helps users deepen their understanding of health-related issues. Furthermore, the results of specific health checkups for National Health Insurance subscribers can also be automatically synchronized with Asmile, allowing them to check their health status. Another feature of the Asmile app is that the ranking of cumulative annual points among all users, those in the same region, and those of the same sex and age group should encourage individuals to be aware of their position within the community and foster a sense of competition, which may increase their motivation to improve health awareness [23].

Using data from the specific health checkups of individuals insured by the National Health Insurance of Osaka Prefecture, our research group developed artificial intelligence models to predict the probability of the onset of diabetes, hypertension, and dyslipidemia within 3 years. By applying a machine learning approach, these models demonstrated high accuracy in predicting the probabilities of disease onset [24]. The artificial intelligence models were implemented in the Asmile app in December 2021, allowing users to know their future disease risk. Therefore, using Asmile is expected to increase awareness of one’s health and promote various health activities, such as increased physical activity. In addition, other previous studies that used the Asmile app have revealed the impact of the declaration of a state of emergency for COVID-19 on smoking behavior and the relationship between oral frailty and falls [25,26].

### Participants

The participants were new users registered for Asmile between fiscal years (FYs) 2020 and 2023. We excluded participants who had missing information on date of birth or sex, whose current address was not in Osaka Prefecture, or who were younger than 20 years or older than 80 years at the time of registration. We also excluded participants whose step count data were not linked for 35 days before registration to 28 days after registration.

### Location, Weather, and Population in Osaka Prefecture

Osaka Prefecture in Japan is located at approximately 135° longitude and 35° latitude, situated in the Kansai region, near the geographic center of the Japanese archipelago. Japan experiences a temperate monsoon climate with 4 distinct seasons. The rainy season typically lasts from mid-June to mid-July, with increased rainfall in many regions, while typhoons primarily affect Japan from July to October. In Osaka, the summers (June-August) are hot and humid, with temperatures often exceeding 30° C, while winters (December-February) are relatively mild and dry, with minimal snowfall. Another notable characteristic of the city is the heat island effect.

Osaka Prefecture has a population of approximately 8.77 million as of June 1, 2024, making it one of the most populous regions in Japan [27]. The population of those aged 18 years and older who are eligible to use the Asmile app is estimated to be approximately 7.60 million.

### Step Data

The Asmile app records step count data in 2 ways. In the first method, data are automatically transferred from a standard smartphone health care app into Asmile. This method transfers the daily step count for the last 42 days each time the user opens the app. The second method involves manual input of steps recorded by a pedometer. We used only automatically linked data in this study, excluding manual input data. Data for fewer than 200 steps and more than 50,000 steps per day were treated as inappropriate step count data, caused possibly by leaving the smartphone behind or due to system inadequacy; these were treated as missing data. The cutoff values were determined based on the step count distribution of the participants as shown in Multimedia Appendix 1. If different step counts were recorded on iOS and Android for a single day, we considered the larger step count data.

To preprocess the step count data, missing values were imputed using the average of the previous 7 days. After processing the missing data, 80,689 users with no missing step count data for the 28 days before and after registration were included in the analysis.

### Causal Impact

In this study, we used Causal Impact [28] to evaluate the increase in step counts due to new registrations in Asmile. This approach uses a causal inference model based on a Bayesian structural time series model [29] that estimates postintervention counterfactual data from preintervention data. This method estimates intervention effects in time-series data, even in study designs with no control groups.

Let be the actual step data obtained at the time *t*. The date of registration to Asmile is *T*_0_, the preregistration period is *t*=1, …, *T*_0_–1, and the postregistration period is *t*=*T*_0_+1, …, *T*. Causal Impact estimates the counterfactual data after registration {*ŷ*_*T*_0_+1_, …, *ŷ_T_*} if the user does not register from the data before registration {*y*_1_, …, *y*_*T*_0_–1_}. The causal effect of registration was then estimated by comparing the estimated step *ŷ* with the actual step *y* after registration.

The structural time series model can be defined using the following pair of equations:



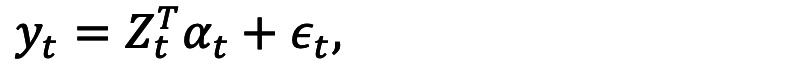





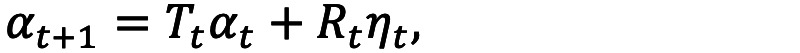



where 
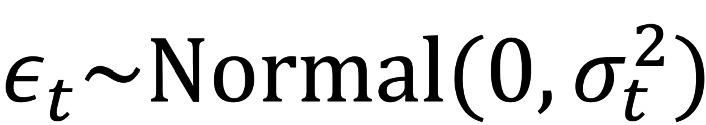
 and *η_t_* ~ Normal(0, *Q_t_*) are independent of all other unknowns. 
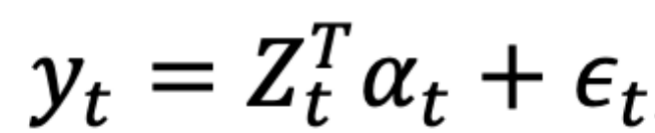
 is the observation equation that links the observed data *y_t_* to a latent *d*-dimensional state vector *α_t_*. *α*_*t*+1_ = *T_t_α_t_* + *R_t_η_t_* is the state equation that governs the evolution of the state vector *α_t_* over time. *Z_t_*, *T_t_*, *R_t_*, and *Q_t_* denote the output vector, transition, control, and state diffusion matrix, respectively. The state *α_t_* includes a local trend and seasonality as follows:



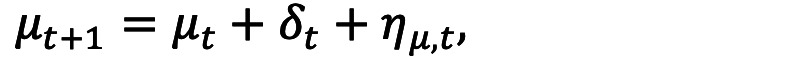





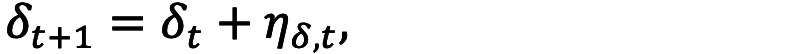





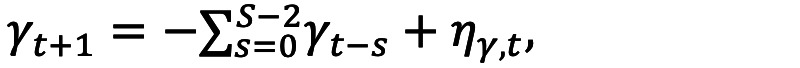



where 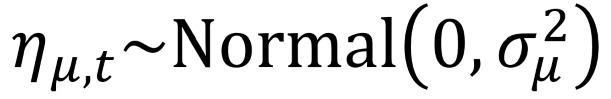 and 
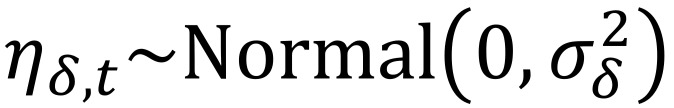
. *µ_t_* denotes the trend value at the time *t*. The *δ_t_* denotes the expected increase in *µ* between times *t* and *t*+1. *S* represents the number of seasons and *γ_t_* denotes their joint contribution to the *y_t_*. The mean of *γ_t_*_+1_ is such that the total seasonal effect is zero when summed over *S* seasons. As the mean step counts were lower on weekends than on weekdays among Asmile users, we set *S*=7 in this study. In addition, previous studies have reported that physical activity is associated with weather conditions, such as temperature, precipitation, and duration of sunshine [30-32]. Therefore, in this study, we included the mean temperature (°C), daily precipitation (mm), sunshine duration (hours), and mean wind speed (m/s) at *t* as covariates. Weather data for Osaka, Osaka Prefecture, Japan, were obtained from the Japan Meteorological Agency website [33].

Next, the intervention’s effect on individual *i* at time *t* is estimated using the following equation:



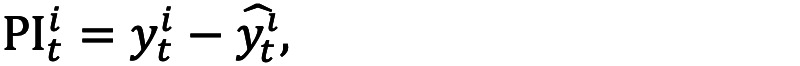



where *t*>*T*_0_. PI is called a pointwise impact. In addition, the cumulative intervention effect 
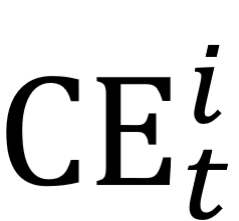
 and average intervention effect 
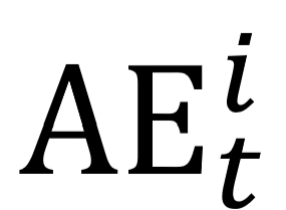
 up to time *t*(*t*>*T*_0_) are defined by the following equations:



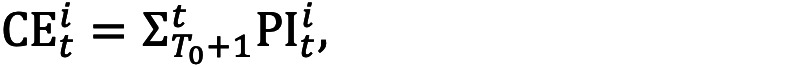





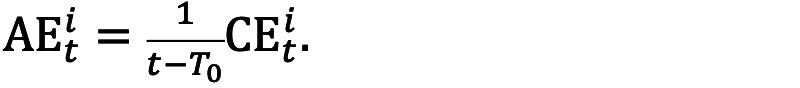



When estimating 
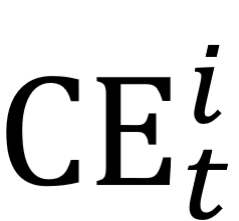
 and 
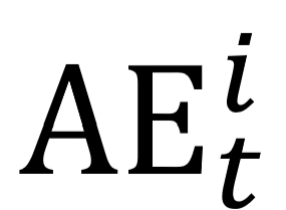
 from all Asmile registrants, the mean values 
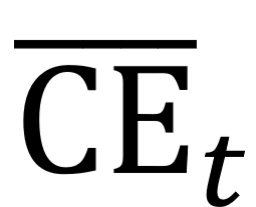
 and 
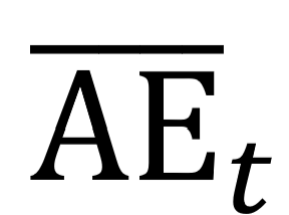
 were obtained. We used the Causal Impact [34] Package in the R software (R Foundation for Statistical Computing) to determine the impact of the estimation. Refer to the studies by Brodersen et al [28] and Scott and Varian [29] for details of the Causal Impact algorithm.

### Statistical Analysis

Continuous variables are expressed as mean (SD) or median (IQR), as appropriate, and categorical variables are expressed as numbers (proportions). The mean step counts before registration were calculated from –28 to –1 days from the registration date, and the mean step counts after registration were calculated from +1 to +28 days from the registration date. A paired *t* test was used to compare the mean step counts before and after the registration date. The effect of registration for Asmile on step count was estimated using the Causal Impact and 
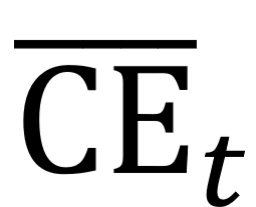
 and 
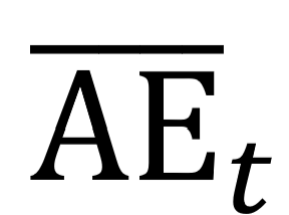
 were obtained from the mean of all users.

Stratified analyses were performed based on sex, age, mean step count before registration, and registration period, categorized by FY (2020-2023) and season: spring (March-May), summer (June-August), fall (September-November), and winter (December-February). For sensitivity analysis, the effect of registration for Asmile was estimated for 20,511 individuals with complete step data. In addition, to demonstrate the effect of increasing step count over a longer period after the Asmile registration, we conducted similar analyses on participants who could be followed up to 56 days, 84 days, and 112 days after registration. A *P* value of <.05 was considered statistically significant. All analyses were performed using R (version 4.2.2; R Foundation for Statistical Computing) [35].

### Ethical Considerations

The study protocol was approved by the ethics committee of the Health and Counseling Center of The University of Osaka (institutional review board approval number 8 in 2024). All procedures involving human participants were conducted according to the 1964 Declaration of Helsinki and its later amendments or comparable ethical standards. At the time of app registration, the Asmile users consented to the use of nonidentifiable information in accordance with the terms of service related to the privacy policy. Informed consent to this study was not obtained from the participants because all data were anonymized according to the Japanese Ethical Guidelines for Medical and Health Research Involving Human Subjects enacted by Japan’s Ministry of Health, Labor, and Welfare. Anonymized data were provided by the Osaka Prefectural Government, and no compensation was provided to the participants.

## Results

### Characteristics

**Figure 1:**
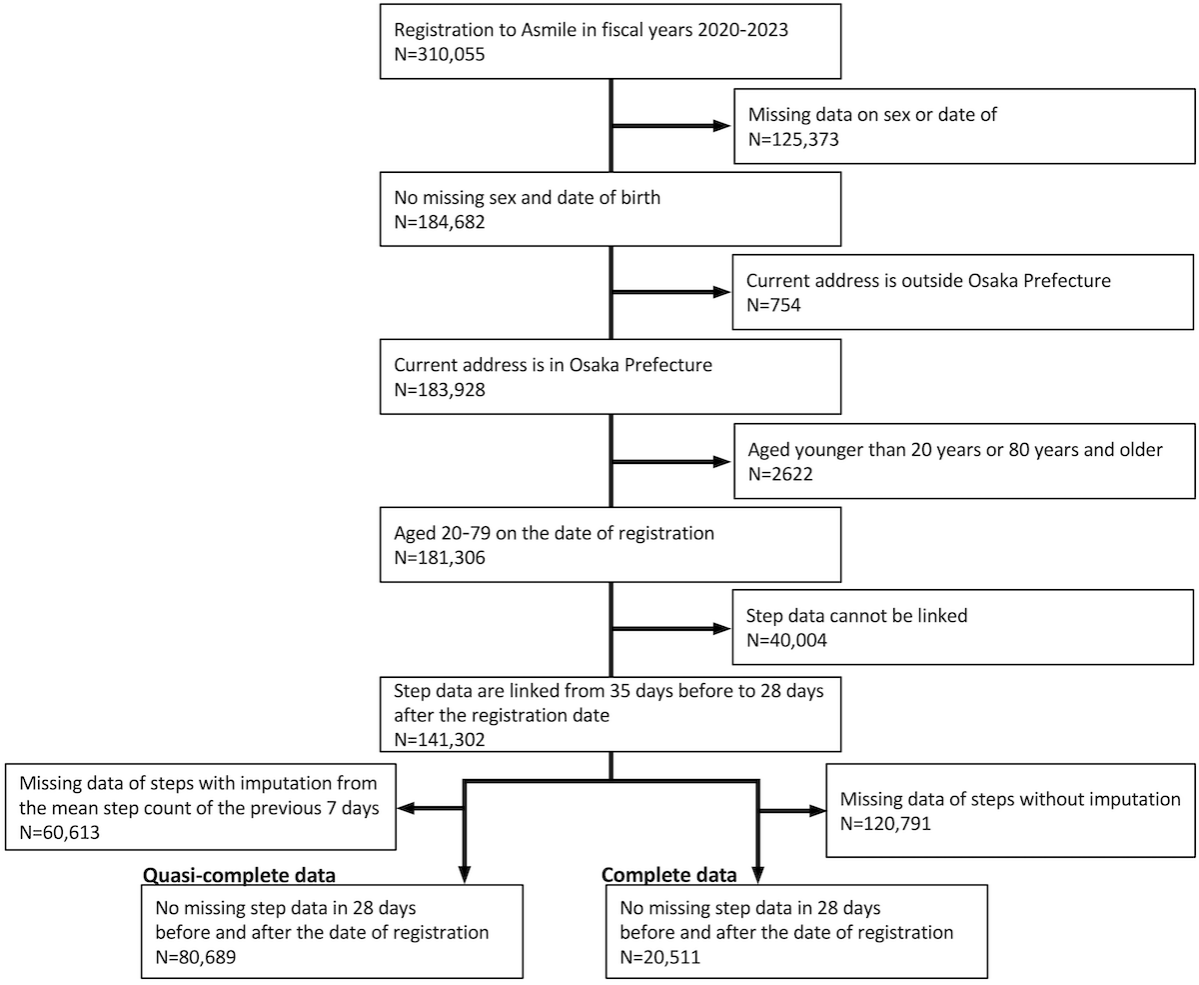
A flow diagram of study entry.

The study included 310,055 users newly registered with Asmile between FY2020 and FY2023. Of these, 80,689 were included in the analysis, excluding 125,373 users who lacked information on sex or date of birth, 754 users whose current address was not Osaka Prefecture, 2622 users younger than 20 years or 80 years and older, 40,004 users whose step count data for the analysis period could not be linked, and 60,613 users whose preprocessed step count data were missing for 28 days before and after registration (Figure 1). The total number of participants with complete data was 20,511.

Table 1 shows the baseline characteristics of the 80,689 users. Of the participants, 38.5% (31,082/80,689) were men and 61.5% (49,607/80,689) were women, with a mean age of 51.6 (SD 13.2) years. The largest proportion of new members was registered in FY2020, and the largest number of new registrations occurred in the spring. The mean step counts before registration were 6034.9 (3978.4-6381.4) steps per day for men and 4364.1 (2808.8-6381.4) steps per day for women. The baseline characteristics of the complete data of the 25,011 participants are shown in Multimedia Appendix 2.

**Table 1:** Baseline characteristics.

Characteristics		Overall (N=80,689)	Men (n=31,082)	Women (n=49,607)
**Age (years), mean (SD)**		51.6 (13.2)	53.3 (13.2)	50.6 (13.1)
	20-29	5384 (6.7)	1650 (5.3)	3734 (7.5)
	30-39	9770 (12.1)	3307 (10.6)	6463 (13.0)
	40-49	19,083 (23.7)	6822 (21.9)	12,261 (24.7)
	50-59	21,305 (26.4)	7791 (25.1)	13,514 (27.2)
	60-69	18,237 (22.6)	8174 (26.3)	10,063 (20.3)
	70-79	6910 (8.6)	3338 (10.7)	3572 (7.2)
**Fiscal year, n (%)**				
	2020	34,491 (42.7)	12,496 (40.2)	21,995 (44.3)
	2021	12,946 (16.0)	5097 (16.4)	7849 (15.8)
	2022	18,652 (23.1)	7503 (24.1)	11,149 (22.5)
	2023	14,600 (18.1)	5986 (19.3)	8614 (17.4)
**Season, n (%)**				
	Spring	38,389 (47.6)	14,055 (45.2)	24,334 (49.1)
	Summer	13,311 (16.5)	5452 (17.5)	7859 (15.8)
	Fall	16,604 (20.6)	6486 (20.9)	10,118 (20.4)
	Winter	12,385 (15.3)	5089 (16.4)	7296 (14.7)
Mean steps before registration without imputation, median (IQR)		4964 (3139-7234)	6035 (3978-8552)	4364 (2809-6381)
Mean steps before registration with imputation, median (IQR)		4957 (3134-7232)	6032 (3971-8553)	4362 (2804-6380)
Mean steps after registration without imputation, median (IQR)		5411 (3551-7683)	6511 (4397-9021)	4834 (3203-6803)
Mean steps after registration with imputation, median (IQR)		5403 (3547-7681)	6506 (4389-9019)	4830 (3194-6807)

### Effects of Registration on Step Count

**Figure 2:**
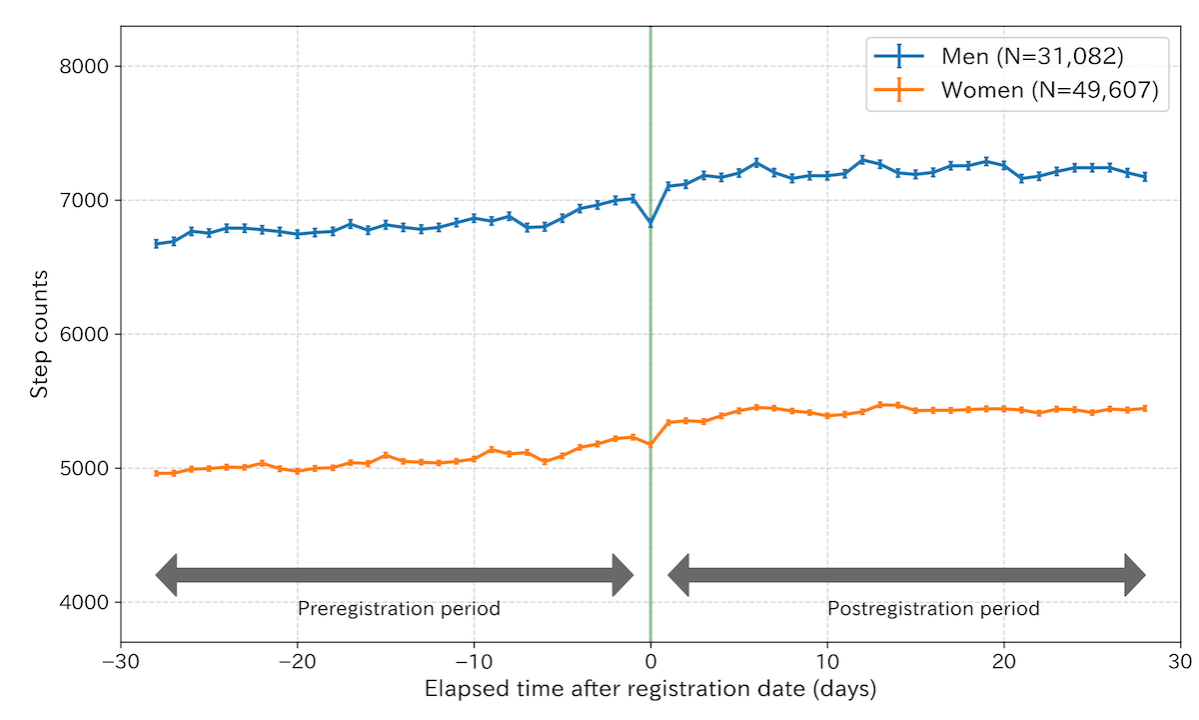
Average step count of 80,689 Asmile users by sex. The horizontal axis represents the elapsed days after registration dates, and the vertical axis shows the mean steps per day. The green line indicates the registration date for each user. Error bars indicate standard errors.

Figure 2 shows the changes in mean step counts before and after Asmile registration. The mean step counts for men 28 days before and after Asmile registration were 6034.9 steps per day and 6510.8 steps per day, respectively. For women, the mean step count before and after registration was 4364.1 steps per day and 4833.6, respectively. A pre- and postregistration comparison of the mean step counts over 4 weeks showed statistically significant increases of 475.9 steps per day (*P*<.001) for men and 469.5 steps per day (*P*<.001) for women. In addition, an increasing tendency in step counts was observed 1 week before the registration date. Multimedia Appendix 3 plots the step counts stratified by age, registration period, and the mean preregistration step count. Table 2 shows the average step counts before and after registration stratified by sex and age group. For both men and women, there were statistically significant increases in step counts in all age groups after registration for Asmile. In addition, in age groups such as those in their 60s and 70s, the average step counts before registration exceeded the daily target step counts defined in the Asmile app.

**Figure 3:**
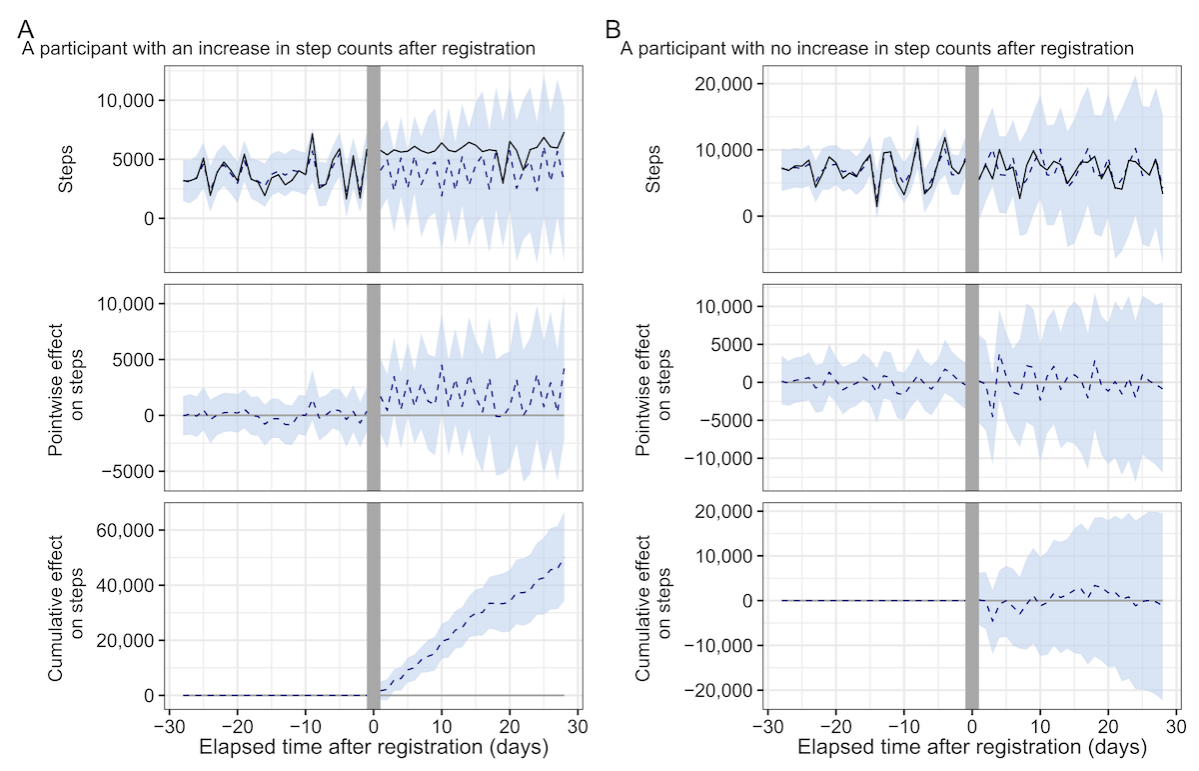
Inferring results of the effect on step count by Causal Impact. The horizontal axis represents the number of days elapsed since the registration date, and the vertical axis represents the daily step counts. (A) A participant with an increase in step count after registration for Asmile. (B) A participant with no increase in step count after registration for Asmile. (Top) The solid line represents the observed step count, and the dashed line represents the counterfactual estimated result with a 95% credible interval by the Causal Impact. The gray interval represents the registration day and is the period not used for either training or inference. (Middle) Pointwise effect: the difference between the actual and the estimated step counts. (Bottom) Cumulative effect: the cumulative sum of the pointwise impact after registration.

**Figure 4:**
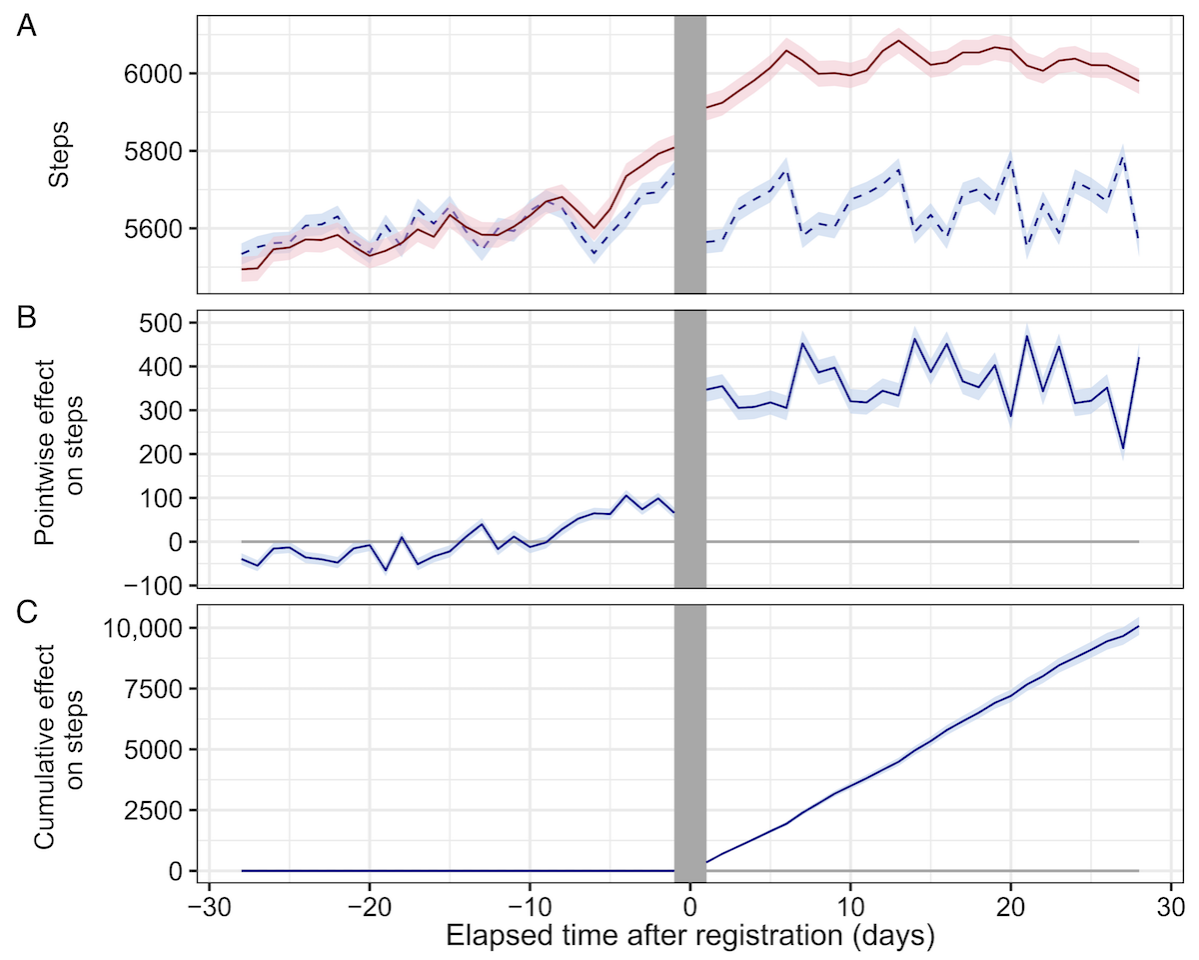
Inferring results of the increase in step count for all participants. (A) The red solid line represents the actual observed mean step count and 95% CI, and the blue dashed line represents the counterfactual estimated mean step count and 95% CI. (B) Mean of pointwise effect and 95% CI. (C) Mean of cumulative effect and 95% CI after registration.

Figure 3 shows examples of the effects of registration on participants estimated by Causal Impact. Figure 3A shows a typical user for whom the increase in step count was caused by Asmile registration, whereas Figure 3B shows a user for whom the increase in step count was not caused by Asmile registration. The effect of an increase in the step count for all participants is shown in Figure 4. Figure 4A shows the actual observed mean step count and the counterfactual estimated mean step count for all participants. For all participants, step-increase effects of 360 steps per day (95% CI 331-389; Figure 4B) and 10,082 steps per 4 weeks (95% CI 9706-10,458; Figure 4C) were obtained.

**Table 2:** Mean step count before and after registration to Asmile and daily target steps for the Asmile app by sex and age.

Gender and age (years)			Participants, N		Mean steps before registration, mean (SD)		Mean steps after registration, mean (SD)		*P* value		Daily target steps
**Male**											
	20-29	1650		7362 (3472)		7956 (3692)		<.001		8000	
	30-39	3307		7232 (3494)		7712 (3439)		<.001		8000	
	40-49	6822		6861 (3791)		7310 (3918)		<.001		7000	
	50-59	7791		6686 (4033)		7107 (4049)		<.001		7000	
	60-69	8174		6503 (4139)		6854 (4197)		<.001		7000 (<65 years); 4000 (≥65 years)	
	70-79	3338		6016 (3682)		6393 (3771)		<.001		4000	
**Female**											
	20-29	3734		5833 (2803)		6340 (2798)		<.001		6000	
	30-39	6463		5059 (2796)		5530 (2824)		<.001		6000	
	40-49	12,261		4832 (2972)		5223 (2968)		<.001		5500	
	50-59	14,514		4819 (3002)		5206 (3048)		<.001		5500	
	60-69	10,063		4883 (3077)		5222 (3082)		<.001		5500 (<65 years); 4000 (≥65 years)	
	70-79	3572		4800 (2953)		5130 (2950)		<.001		4000	

**Figure 5:**
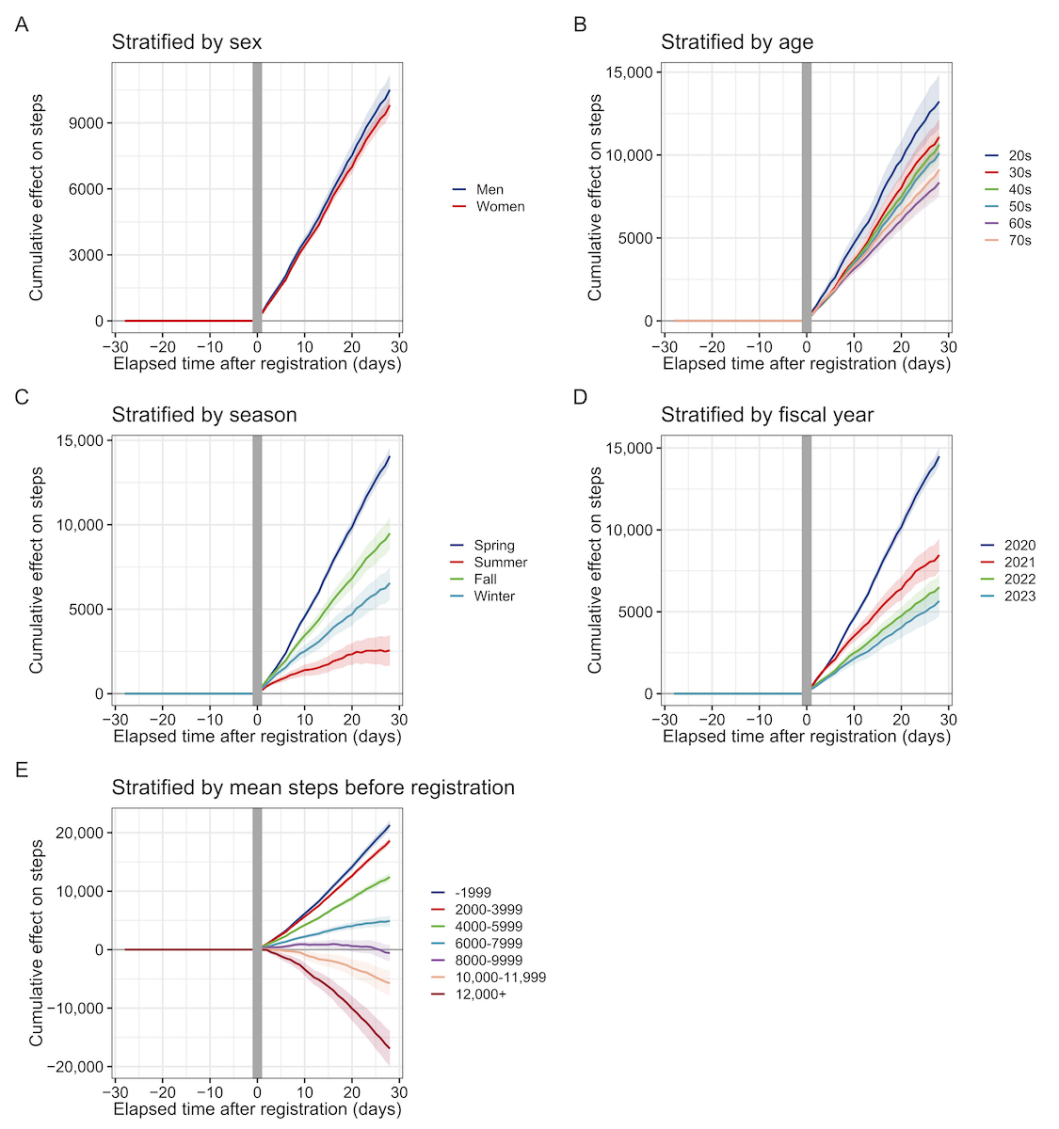
Cumulative effects on step count stratified by baseline characteristics. (A) Stratification by gender (men and women), (B) age group (20s to 70s), (C) season of registration (spring, summer, fall, and winter), (D) fiscal year of registration (2020-2023), and (E) mean daily step count before registration (from <2000 to ≥12,000 steps per day).

Table 3 shows the average effects of step count increases stratified by each baseline characteristic. The results indicate minimal differences between men and women (men: 375, 95% CI 323-428 steps per day; women: 350, 95% CI 316-385 steps per day). The effect on step counts was more pronounced in younger age groups, in those registered in spring or fall, in those registered in FY2020, and in those with lower mean step counts before registration. The cumulative effect curves are depicted in Figure 5, and the cumulative effects are shown in Table 4. The cumulative effects on step counts stratified by baseline characteristics were 10,511 steps per 4 weeks (95% CI 9820-11,203) for men and 9812 steps per 4 weeks (95% CI 9381-10,244) for women (Figure 5A). Similar patterns to the average effects were observed in other stratified groups: by age group (Figure 5B), season of registration (Figure 5C), fiscal year of registration (Figure 5D), and mean step count before registration (Figure 5E).

**Table 3:** Results of all estimates of the average effects of increased step count.

Characteristics			Main analysis^a^													Subanalysis^b^											
			Mean						95% CI						Mean							95% CI					
All			360						331 to 389						302							248 to 356					
**Gender**																											
		Men			375						323 to 428							316						234 to 397			
		Women			350						316 to 385							289						219 to 359			
**Age (years)**																											
		20-29		473						343 to 602							415						168 to 662				
		30-39		396						311 to 482							273						128 to 419				
		40-49		380						320 to 439							314						205 to 422				
		50-59		361						305 to 418							301						193 to 408				
		60-69		298						237 to 359							277						166 to 388				
		70-79		326						232 to 420							308						131 to 484				
**Fiscal year**																											
		2020				518						476 to 560							405						320 to 489		
		2021				302						226 to 379							261						129 to 394		
		2022				232						169 to 295							237						132 to 343		
		2023				202						128 to 276							229						101 to 357		
**Season**																											
		Spring						503						463 to 543							416						338 to 494
		Summer						91						20 to 163							64						–63 to 191
		Fall						339						271 to 407							322						207 to 437
	Winter						234						152 to 316							217						71 to 362	
**Baseline steps**																											
	–1999					762						707 to 816							779						608 to 950		
	2000 to 3999					665						623 to 708							665						576 to 755		
	4000 to 5999					443						390 to 497							509						416 to 602		
	6000 to 7999					175						101 to 249							249						138 to 361		
	8000 to 9999					–21						–136 to 93							102						–54 to 258		
	10,000 to 11,999					–206						–381 to 31							–55						–284 to 174		
	12,000+					–604						–840 to 369							–442						–717 to 166		

^a^The analysis includes 80,689 users with quasi-complete step data.

^b^The analysis includes 20,511 users with complete step data.

**Table 4:** Results of all estimates of the cumulative effects of increased step count.

Characteristics		Main analysis^a^						Subanalysis^b^			
		Mean				95% CI		Mean	95% CI		
All		10,082				9706 to 10,458		8460	7807 to 9112		
**Gender**											
	Men		10,511		9820 to 11,203		8838			7835 to 9841	
	Women		9812		9381 to 10,244		8088			7251 to 8925	
**Age (years)**											
	20-29		13,235			11,611 to 14,860		11,620			8715 to 14,524
	30-39		11,096			9985 to 12,207		7657			5864 to 9450
	40-49		10,632			9871 to 11,393		8778			7471 to 10,085
	50-59		10,120			9407 to 10,832		8416			7103 to 9730
	60-69		8346			7552 to 9140		7766			6434 to 9098
	70-79		9136			7886 to 10,386		8612			6366 to 10,858
**Fiscal year**											
	2020		14,497	13,949 to 15,045				11,328			10,293 to 12,362
	2021		8466	7455 to 9476				7319			5650 to 8989
	2022		6499	5725 to 7273				6644			5382 to 7905
	2023		5661	4746 to 6577				6403			4901 to 7905
**Season**											
	Spring		14,080	13,571 to 14,590				11,651			10,719 to 12,583
	Summer		2561	1658 to 3463				1791			233 to 3348
	Fall		9497	8550 to 10,445				9017			7508 to 10,526
	Winter		6554	5600 to 7509				6068			4477 to 7659
**Baseline steps**											
	–1999		21,322		20,477 to 22,167		21,809			19,233 to 24,385	
	2000-3999		18,633		18,061 to 19,206		18,623			17,469 to 19,778	
	4000-5999		12,417		11,746 to 13,089		14,245			13,119 to 15,370	
	6000-7999		4906		4009 to 5803		6983			5681 to 8286	
	8000-9999		–594		–1979 to 790		2854			1082 to 4626	
	10000-11999		–5767		–7891 to –3642		–1536			–4290 to 1218	
	12000+		–16,923		–19,962 to –13,884		–12,368			–15,711 to –9025	

^a^The analysis includes 80,689 users with quasi-complete step data.

^b^The analysis includes 20,511 users with complete step data.

### Sensitivity Analysis

For the sensitivity analysis, the effect of registration on step counts was estimated for 25,011 participants with complete step data 28 days before and after the registration date. The increases in step counts were 302 steps per day (95% CI 248-356) and 8460 steps per 4 weeks (95% CI 7807-9112) in 25,011 participants (Tables 3 and 4). Further sensitivity analyses were conducted on participants who could be followed up to 56, 86, and 112 days after registration. The number of participants was 67,496 at 56 days, 57,719 at 84 days, and 50,504 at 112 days. The baseline characteristics are shown in Multimedia Appendix 4. The average effects of increased step count estimated by Causal Impact are shown in Table 5. Although the number of participants decreased with longer follow-up periods, the effect of increasing step count became smaller with longer follow-up periods: 338 steps per day (95% CI 304-372) at 56 days, 300 steps per day (95% CI 260-341) at 84 days, and 275 steps per day (95% CI 230-320) at 112 days. The cumulative effects are shown in Multimedia Appendix 5.

**Table 5:** Results of all estimates of the average effects of increased step count at 56, 84, and 112 days of follow-up.

Characteristics		56 days			84 days			112 days	
		Mean	95% CI	Mean		95% CI	Mean		95% CI
All		338	304 to 372	300		260 to 341	275		230 to 320
**Gender**									
	Men	344	283 to 406	309		236 to 382	297		216 to 378
	Women	334	294 to 374	295		247 to 343	261		209 to 314
**Age (years)**									
	20-29	408	248 to 568	320		120 to 519	319		92 to 545
	30-39	343	237 to 449	285		155 to 416	252		104 to 399
	40-49	372	301 to 442	345		259 to 431	317		220 to 414
	50-59	366	300 to 431	329		251 to 407	308		221 to 394
	60-69	266	196 to 335	240		160 to 321	214		127 to 301
	70-79	312	209 to 415	280		161 to 399	261		135 to 388
**Fiscal year**									
	2020	527	476 to 578	472		410 to 535	419		348 to 489
	2021	261	175 to 346	238		140 to 336	245		140 to 349
	2022	196	126 to 266	170		88 to 252	159		71 to 247
	2023	202	118 to 287	205		105 to 305	209		96 to 322
**Season**									
	Spring	510	462 to 558	450		392 to 508	358		294 to 422
	Summer	59	–22 to 140	114		20 to 209	220		116 to 323
	Fall	242	163 to 321	144		52 to 237	107		8 to 205
	Winter	279	187 to 371	330		225 to 435	378		257 to 500
**Baseline steps**									
	≥1999	904	837 to 972	981		899 to 1063	1049		957 to 1140
	2000-3999	746	696 to 796	776		716 to 836	796		728 to 864
	4000-5999	452	391 to 514	434		360 to 507	408		328 to 488
	6000-7999	113	30 to 196	59		–38 to 157	34		–71 to 139
	8000-9999	–126	–254 to 3	–174		–323 to –24	–196		–357 to –34
	10,000-11,999	–336	–531 to –140	–448		–673 to –224	–494		–736 to –252
	12,000+	–847	–1107 to –588	–989		–1277 to –701	–1068		–1374 to –761

## Discussion

### Principal Findings

In this observational study of 80,689 Asmile users aged 20-79 years, it was confirmed that registering for the Asmile app increased step count by approximately 360 steps per day and approximately 10,082 steps in 4 weeks. The stratified analysis showed that the increase in step count was more significant in the younger age group. The intervention had a more substantial effect on the group that had taken fewer step counts before registration. It was also confirmed that the effects were higher in the spring, fall, and FY2020 groups.

This study conducted a subgroup analysis based on the baseline characteristics. First, the results of comparing the effect of the mHealth app on step count by sex showed almost no differences. This finding suggests that the impact of the mHealth app on step count is significant, regardless of sex. Next, the results of the subgroup analysis by age showed that increasing step count was more important in the younger age group, and the effect of registration in the mHealth app was lesser in the older age group. One reason for this is the influence of the target steps set in the Asmile app. The Asmile app aims to promote healthy activities, with the target steps set at 8000 for men younger than 40 years, 7000 for those aged 40-65 years, and 4000 for those aged 65 years and older. For women, the target was 6000 steps for those younger than 40 years, 5500 for those aged 40-65 years, and 4000 for those aged 65 years and older. By achieving these targets, users are awarded points to participate in a lottery within the app. In the 28 days before registration, the average step count taken by men aged 20-29 years was 7362 (SD 3472) steps per day, and the average step count taken by women was 5833 (SD 2803) steps per day. In comparison, the average step count of men aged 70-79 was 6016 (SD 3682) steps per day, and the average step count of women was 4800 (SD 2953) steps per day. Although the average step count was lower for the older adults than for the young, the average step count of the older adults before registration on the Asmile app had already exceeded the target steps for their age group (Table 2). Therefore, older adults may increase step counts further by setting higher target steps.

Stratified analysis by registration season showed that the effect of registration on step count was more significant in the groups who registered in spring or fall and less significant in summer or winter. This is because in Japan, the temperatures in summer and winter are not suitable for exercise, whereas walking is more accessible in spring and fall. The decrease in average steps in winter and summer was also observed in an analysis conducted in Yokohama, Japan, which examined the relationship between monthly average steps and temperature [30]. Especially in the spring, the increase in step counts was the greatest than that in other seasons due to the start of a new FY, known as the “fresh start effect.” In contrast, the step counts did not increase during January, the New Year’s period. In Japan, many schools and businesses are closed during this time, which may explain why people take fewer steps to spend more time at home.

The results of the stratified analysis by FY of registration show that the effect of the increase in step count for the group registered in FY2020 was greater than that in other years. This effect may be because of the worldwide coronavirus pandemic. In Japan, on April 7, 2020, following an increase in COVID-19 cases, the government declared a state of emergency in 7 major prefectures, including Osaka, which was extended to all other prefectures on April 16 [36]. Under the state of emergency, prefectural governors could order residents to stay at home without penalties for noncompliance. During this period, schools were closed, and teleworking became more common. However, the Japanese government could not force the closure of private businesses or impose strict stay-at-home orders. Meanwhile, outdoor activities such as walking and jogging were allowed and specifically not restricted during this period [37]. It is believed that the declaration of a state of emergency in Osaka Prefecture led to changes in the lifestyle of many people. As a result, individuals who previously had limited opportunities to walk owing to work or school may have had more opportunities to increase their physical activity. However, several previous studies have reported that physical activity levels decreased during the COVID-19 pandemic [38-40]. While it remains unclear whether the increase in step counts was specific to Asmile users, further investigation is needed.

When we focused on the mean step count before registration, the effect of registration on the step count tended to be greater for those with fewer steps. In particular, the group whose mean step count was less than 2000 steps per day before registration for Asmile showed an increase of more than 20,000 steps per 4 weeks after registration. In contrast, the groups with step count of more than 10,000 steps per day before registration showed a significant decrease in step count after registration. Although there is insufficient evidence for the effect of step reduction on populations with high baseline step count, previous studies have reported that the dose-response association of step count showed that the risk of all-cause mortality and diabetes in older adults is lowest at approximately 8000 steps per day [41,42]. A cohort study of 661,137 men and women showed similar L-shaped curves for physical activity and all-cause mortality [43]. This app may help increase step count for populations with low daily and moderate step count for those who exercise excessively.

This study primarily investigated the short-term effect of step count 28 days after registration. An additional question of interest was how long this effect persisted. To address this, we performed similar analyses on participants who could be followed up to 56, 84, and 112 days after registration. The results showed that the effect of the increase in step count diminished as the follow-up period extended, suggesting that the effect of registering with Asmile may be temporary. However, since the analysis was limited to participants whose step counts were recorded for up to 112 days after registration, the results should be interpreted cautiously. Further research is needed to investigate the long-term sustainability of the effect of Asmile registration.

### Comparison With the Literature

Several previous studies have evaluated the impact of mHealth apps on physical activity; however, most had limited sample sizes. In a systematic review, Yerrakalva et al [14] reported the impact of an mHealth app intervention on physical activity in adults aged 55 years and older. Their meta-analysis included 486 participants (324/486, 66.7% women; mean age of 68 years) and found that the mHealth app increased step count by 506 steps per day (95% CI –80 to 1092). These results were similar to our interventions, although they were not statistically significant.

Hamaya et al [16] evaluated the impact of physical activity levels on CVD risk factors using an mHealth app tied to Japanese insurance and health checkup databases. Their analysis showed that 12,602 users experienced an increase of 510 steps per day after using the mHealth app. Compared with our results, the increase in step count was slightly more significant in the results of Hamaya et al [16]. Their analysis was based on a comparison of average step counts over a year before and after app registration. Their approach may have limited their analysis to highly health-conscious participants who were committed enough to continue using the app for approximately 1 year and were more likely to achieve increased step counts. In contrast, our analysis focused on the short-term effect of step count increases within 4 weeks after app registration. This approach may have provided a more generalizable estimate of the step count increase effect associated with health promotion app registration. In addition, our results showed increased step counts as early as the day after registration in Asmile. This suggests that Asmile’s unique feature of providing daily incentives based on daily health activities amplified the short-term effect on step count increase. In addition, their study used a simple pre- and postcomparison of the mean step count because they did not include a control group. Although no control group was available for our analysis, the intervention effects were estimated using the Causal Impact from the intervention group only. This method allowed for assessing the effect of daily and 4-week increases in step count with an appropriately estimated counterfactual control group.

### Benefits of Increasing Step Count

A systematic review conducted by Hall et al [44] reported that an increase of 1000 steps per day reduced all-cause mortality by 6%-36% and CVD risk by 5%-21% in adults. According to Kraus et al [45], an increase of 2000 steps per day reduced the risk of developing diabetes in a randomized controlled trial of 9306 individuals with impaired glucose tolerance. Igarashi et al [46] showed in a systematic review that an increase of 2000 steps per day resulted in a systolic blood pressure reduction in the risk of developing diabetes.

In addition, Banach et al [47] showed in a meta-analysis that an increase of 1000 steps per day reduced all-cause mortality by 15%, and an increase of 500 steps per day reduced the risk of CVD mortality by 7%. In addition, Kraus et al [45] showed that an increase of 500 steps per day reduced the CVD incidence by 11% in older people with exercise limitations. These results suggest that increased step counts by mHealth apps such as Asmile can improve health.

### Strengths and Limitations

The strengths of this study include the large sample size, the ability to estimate intervention effects on step count stratified by several baseline characteristics, and the use of Causal Impact to assess the effect on step count from the intervention group only. However, this study has some limitations.

First, because a control group that did not use the app was unprepared, the exact causal effect could be estimated only through a randomized controlled trial. However, it took much work to include such a large number of participants, and the Hawthorne effect may have overestimated the increase in step count in the app-using group. In this sense, this study is more likely to demonstrate the real impact of increased step count. However, this analysis cannot determine whether using the app improved health awareness or whether health awareness had already increased before using the app. Second, the members of this app are likely to be highly health conscious, and caution is needed when generalizing and interpreting the results of this analysis. The participants of this analysis are users who could continuously record their steps and were likely to have a relatively high level of health awareness. Another area for improvement is that we could not obtain health status and hospital attendance on the date of Asmile registration. These are essential unobserved confounders because behavioral changes and physical status are closely related. In future studies, it will be necessary to consider the baseline characteristics of users who use items from specific health checkups. Third, the Causal Impact algorithm estimates counterfactual data after registration based on data from before the Asmile registration, which means that the accuracy of the estimated step count increase effect depends on the precision of the prediction of the counterfactual data. In this analysis, owing to the limitations of the app, only data from up to 28 days before the Asmile registration date were available for prediction. Using a longer period of data for prediction can improve the accuracy. In addition, the Causal Impact algorithm cannot account for other events after registration that may influence changes in step count. Fourth, this analysis is limited to examining the short-term effect of step count increases, and it does not demonstrate how long the step count increase effect persists. Addressing this issue requires considering long-term step count fluctuations due to seasonal variations, which we aim to explore in future research. Fifth, the accuracy of step measurement using smartphones is a potential limitation. Previous studies have suggested that step counts measured by smartphones may be underestimated [48-50]. However, since this analysis focused on the increase in step counts, we believe that the impact of smartphone underestimation is minimal.

### Conclusions

This study showed that using the mHealth app “Asmile” increased step counts by approximately 360 steps per day and approximately 10,082 steps over 4 weeks. Increased physical activity is expected to help improve health conditions, such as weight loss and reducing CVD risk.

## Appendix

Multimedia Appendix 1 Distribution of step counts for the participants.

Multimedia Appendix 2 Baseline characteristics for 25,011 with complete data.

Multimedia Appendix 3 Average step count stratified by baseline characteristics. Error bars indicate standard errors.

Multimedia Appendix 4 Baseline characteristics of participants by follow-up duration.

Multimedia Appendix 5 Estimates of cumulative effects of increased step count by follow-up duration.

## Abbreviations

CVD: cardiovascular disease
DID: difference-in-differences
FY: fiscal year
mHealth: mobile health

## Data Availability

Data cannot be shared publicly because local governments own medical checkup data. Data are available from the Health and Counseling Center, The University of Osaka (contact via campuslifekenkou-syomu@office.osaka-u.ac.jp) for researchers who meet the criteria for access to confidential data.
