# Supplementary material for: Effects of Mobile Health Care App "Asmile" on Physical Activity of 80,689 Users in Osaka Prefecture, Japan: Longitudinal Observational Study
Source: J Med Internet Res. 2025 May 21;27:e65943. doi: 10.2196/65943 (PMC12138302; doi:10.2196/65943)
Supplement: Multimedia Appendix 1 [file jmir_v27i1e65943_app1.docx]

Multimedia Appendix 1

Distribution of step counts for the participants.

| count | mean | std | min | 5% | 10% | 25% | 50% | 75% | 90% | 95% | max |
| --- | --- | --- | --- | --- | --- | --- | --- | --- | --- | --- | --- |
| 5,072,425 | 5,560 | 6,054 | 1 | 122 | 496 | 1,797 | 4,459 | 7,969 | 11,793 | 14,565 | 3,905,543 |

Histograms of step counts for the participants.

(Top) x-axis: Step counts (×10,000), Range = [0, 100,000]; y-axis: Frequency (log scale). (Bottom) x-axis: Step counts, Range = [0, 1,000]; y-axis: Frequency.

|  |
| --- |
|  |
