# Supplementary material for: Effects of Mobile Health Care App "Asmile" on Physical Activity of 80,689 Users in Osaka Prefecture, Japan: Longitudinal Observational Study
Source: J Med Internet Res. 2025 May 21;27:e65943. doi: 10.2196/65943 (PMC12138302; doi:10.2196/65943)
Supplement: Multimedia Appendix 2 [file jmir_v27i1e65943_app2.docx]

Multimedia Appendix 2

Baseline characteristics for 25,011 with complete data.

|  | Overall | Men | Women |
| --- | --- | --- | --- |
| n | 25,011 | 12,400 | 12,611 |
| Age, mean (SD) | 51.5 (13.1) | 52.7 (13.1) | 50.4 (13.0) |
| 20–29 | 1471 (5.9) | 603 (4.9) | 868 (6.9) |
| 30–39 | 3333 (13.3) | 1493 (12.0) | 1840 (14.6) |
| 40–49 | 6075 (24.3) | 2938 (23.7) | 3137 (24.9) |
| 50–59 | 6384 (25.5) | 3070 (24.8) | 3314 (26.3) |
| 60–69 | 5687 (22.7) | 3090 (24.9) | 2597 (20.6) |
| 70–79 | 2061 (8.2) | 1206 (9.7) | 855 (6.8) |
| Fiscal Year, n (%) |  |  |  |
| 2020 | 9332 (37.3) | 4678 (37.7) | 4654 (36.9) |
| 2021 | 4295 (17.2) | 2149 (17.3) | 2146 (17.0) |
| 2022 | 6448 (25.8) | 3146 (25.4) | 3302 (26.2) |
| 2023 | 4936 (19.7) | 2427 (19.6) | 2509 (19.9) |
| Season (%) |  |  |  |
| Spring | 10978 (43.9) | 5438 (43.9) | 5540 (43.9) |
| Summer | 4301 (17.2) | 2153 (17.4) | 2148 (17.0) |
| Fall | 5740 (22.9) | 2739 (22.1) | 3001 (23.8) |
| Winter | 3992 (16.0) | 2070 (16.7) | 1922 (15.2) |
| Mean steps before registration  without imputation (median [IQR]) | 6290.8  [4311.5, 8722.8] | 7041.3  [5000.3, 9593.5] | 5601.9  [3781.0, 7746.9] |
| Mean steps before registration  with imputation (median [IQR]) | 6290.8  [4311.5, 8722.8] | 7041.3  [5000.3, 9593.5] | 5601.9  [3781.0, 7746.9] |
| Mean steps after registration  without imputation (median [IQR]) | 6670.2  [4659.9, 9078.8] | 7416.2  [5323.7, 9963.2] | 5979.5  [4154.8, 8120.0] |
| Mean steps after registration  with imputation (median [IQR]) | 6670.2  [4659.9, 9078.8] | 7416.2  [5323.7, 9963.2] | 5979.5  [4154.8, 8120.0] |

SD, standard deviation; IQR, interquartile range.
