# Supplementary figures and images for: Effects of Mobile Health Care App "Asmile" on Physical Activity of 80,689 Users in Osaka Prefecture, Japan: Longitudinal Observational Study
Source: J Med Internet Res. 2025 May 21;27:e65943. doi: 10.2196/65943 (PMC12138302; doi:10.2196/65943)

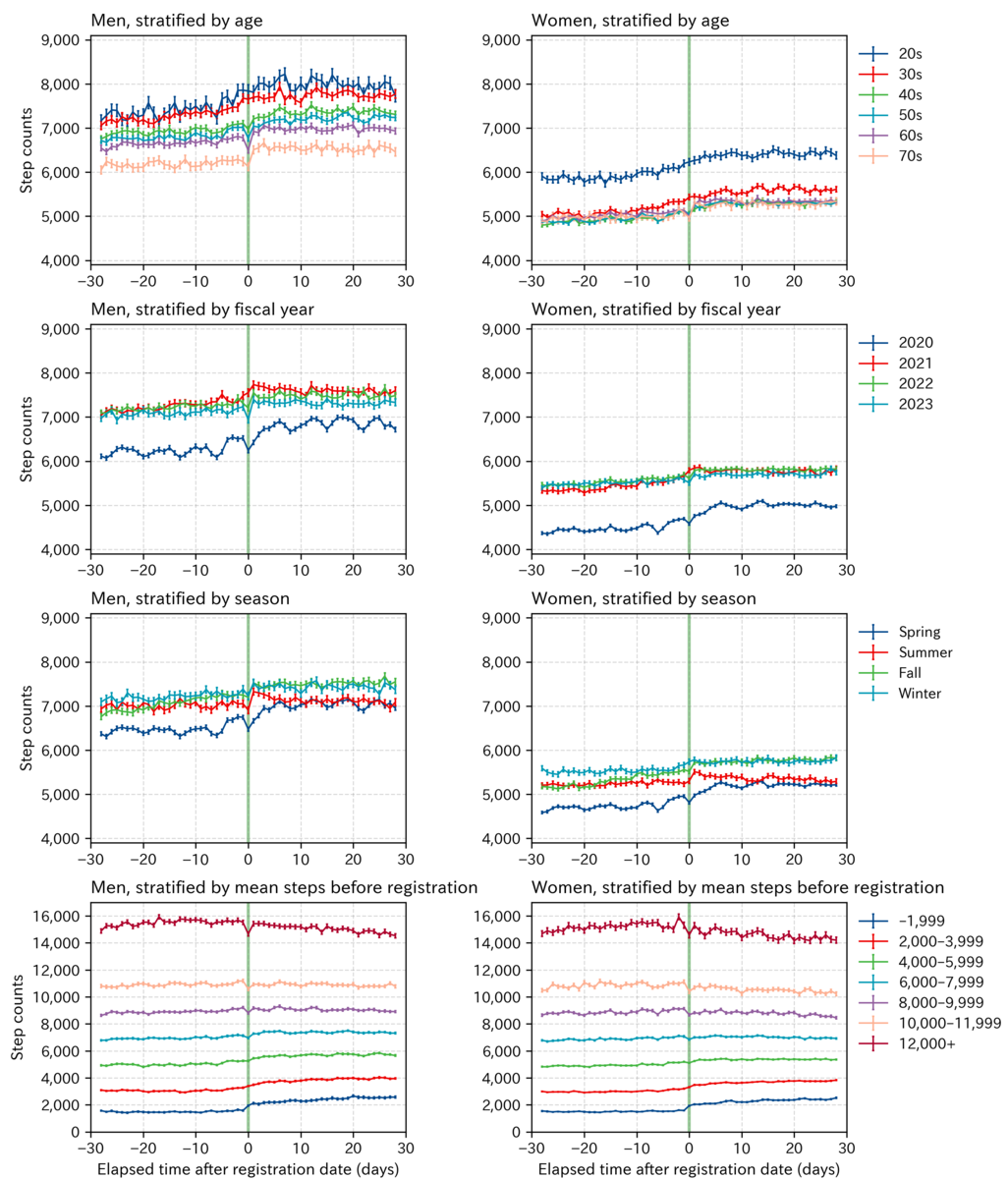

Supplement: Multimedia Appendix 3 [file jmir_v27i1e65943_app3.png]
