# Supplementary material for: Effects of Mobile Health Care App "Asmile" on Physical Activity of 80,689 Users in Osaka Prefecture, Japan: Longitudinal Observational Study
Source: J Med Internet Res. 2025 May 21;27:e65943. doi: 10.2196/65943 (PMC12138302; doi:10.2196/65943)
Supplement: Multimedia Appendix 4 [file jmir_v27i1e65943_app4.docx]

Multimedia Appendix 4

Baseline characteristics of participants followed up for 56 days.

|  | Overall | Men | Women |
| --- | --- | --- | --- |
| n | 67,496 | 26,151 | 41,345 |
| Age, mean (SD) | 52.4 (13.2) | 54.1 (13.1) | 51.4 (13.1) |
| 20–29 | 4,054 (6.0) | 1,214 (4.6) | 2,840 (6.9) |
| 30–39 | 7,514 (11.1) | 2,568 (9.8) | 4,946 (12.0) |
| 40–49 | 15,435 (22.9) | 5,597 (21.4) | 9,838 (23.8) |
| 50–59 | 17,914 (26.5) | 6,491 (24.8) | 11,423 (27.6) |
| 60–69 | 16,215 (24.0) | 7,203 (27.5) | 9,012 (21.8) |
| 70–79 | 6,364 (9.4) | 3,078 (11.8) | 3,286 (7.9) |
| Fiscal Year, n (%) |  |  |  |
| 2020 | 26,482 (39.2) | 9,597 (36.7) | 16,885 (40.8) |
| 2021 | 11,517 (17.1) | 4,559 (17.4) | 6,958 (16.8) |
| 2022 | 16,728 (24.8) | 6,766 (25.9) | 9,962 (24.1) |
| 2023 | 12,769 (18.9) | 5,229 (20.0) | 7,540 (18.2) |
| Season, n (%) |  |  |  |
| Spring | 30,506 (45.2) | 11,240 (43.0) | 19,266 (46.6) |
| Summer | 11,436 (16.9) | 4,695 (18.0) | 6,741 (16.3) |
| Fall | 14,591 (21.6) | 5,709 (21.8) | 8,882 (21.5) |
| Winter | 10,963 (16.2) | 4,507 (17.2) | 6,456 (15.6) |
| Mean steps before registration  without imputation, median [IQR] | 4,919  [2,260, 8,414] | 5,929  [2,843, 9,835] | 4,346  [1,982, 7,509] |
| Mean steps before registration  with imputation, median [IQR] | 4,802  [2,258, 8,220] | 5,802  [2,813, 9,664] | 4,262  [2,000, 7,317] |
| Mean steps after registration  without imputation, median [IQR] | 5,122  [3,253, 7,423] | 6,235  [4,123, 8,772] | 4,521  [2,912, 6,539] |
| Mean steps after registration  with imputation, median [IQR] | 5,116  [3,245, 7,420] | 6,230  [4,119, 8,771] | 4,513  [2,903, 6,537] |

SD, standard deviation; IQR, interquartile range.

Baseline characteristics of participants followed up for 84 days.

|  | Overall | Men | Women |
| --- | --- | --- | --- |
| n | 57,719 | 22,444 | 35,275 |
| Age, mean (SD) | 53.0 (13.1) | 54.6 (13.0) | 52.0 (13.1) |
| 20–29 | 3,192 (5.5) | 953 (4.2) | 2,239 (6.3) |
| 30–39 | 6,088 (10.5) | 2,090 (9.3) | 3,998 (11.3) |
| 40–49 | 12,771 (22.1) | 4,636 (20.7) | 8,135 (23.1) |
| 50–59 | 15,281 (26.5) | 5,530 (24.6) | 9,751 (27.6) |
| 60–69 | 14,585 (25.3) | 6,446 (28.7) | 8,139 (23.1) |
| 70–79 | 5,802 (10.1) | 2,789 (12.4) | 3,013 (8.5) |
| Fiscal Year, n (%) |  |  |  |
| 2020 | 21,069 (36.5) | 7,653 (34.1) | 13,416 (38.0) |
| 2021 | 10,463 (18.1) | 4,143 (18.5) | 6,320 (17.9) |
| 2022 | 15,239 (26.4) | 6,166 (27.5) | 9,073 (25.7) |
| 2023 | 10,948 (19.0) | 4,482 (20.0) | 6,466 (18.3) |
| Season, n (%) |  |  |  |
| Spring | 24,718 (42.8) | 9,205 (41.0) | 15,513 (44.0) |
| Summer | 10,161 (17.6) | 4,145 (18.5) | 6,016 (17.1) |
| Fall | 13,036 (22.6) | 5,103 (22.7) | 7,933 (22.5) |
| Winter | 9,804 (17.0) | 3,991 (17.8) | 5,813 (16.5) |
| Mean steps before registration  without imputation, median [IQR] | 5,111  [2,361, 8,613] | 6,134  [2,975, 10,078] | 4,521  [2,066, 7,692] |
| Mean steps before registration  with imputation, median [IQR] | 4,990  [2,356, 8,409] | 6,016  [2,929, 9,919] | 4,422  [2,088, 7,503] |
| Mean steps after registration  without imputation, median [IQR] | 5,268  [3,354, 7,580] | 6,409  [4,253, 8,955] | 4,658  [2,996, 6,677] |
| Mean steps after registration  with imputation, median [IQR] | 5,262  [3,347, 7,575] | 6,402  [4,244, 8,949] | 4,652  [2,992, 6,672] |

SD, standard deviation; IQR, interquartile range.

Baseline characteristics of participants followed up for 112 days.

|  | Overall | Men | Women |
| --- | --- | --- | --- |
| n | 50,504 | 19,667 | 30,837 |
| Age, mean (SD) | 53.4 (13.1) | 55.0 (12.9) | 52.4 (13.1) |
| 20–29 | 2,669 (5.3) | 797 (4.1) | 1,872 (6.1) |
| 30–39 | 5,101 (10.1) | 1,750 (8.9) | 3,351 (10.9) |
| 40–49 | 10,860 (21.5) | 3,932 (20.0) | 6,928 (22.5) |
| 50–59 | 13,234 (26.2) | 4,790 (24.4) | 8,444 27.4) |
| 60–69 | 13,303 (26.3) | 5,870 (29.8) | 7,433 (24.1) |
| 70–79 | 5,337 (10.6) | 2,528 (12.9) | 2,809 (9.1) |
| Fiscal Year, n (%) |  |  |  |
| 2020 | 17,502 (34.7) | 6,391 (32.5) | 11,111 (36.0) |
| 2021 | 9,744 (19.3) | 3,831 (19.5) | 5,913 (19.2) |
| 2022 | 14,087 (27.9) | 5,703 (29.0) | 8,384 (27.2) |
| 2023 | 9,171 (18.2) | 3,742 (19.0) | 5,429 (17.6) |
| Season, n (%) |  |  |  |
| Spring | 20,657 (40.9) | 7,736 (39.3) | 12,921 (41.9) |
| Summer | 9,277 (18.4) | 3,770 (19.2) | 5,507 (17.9) |
| Fall | 12,048 (23.9) | 4,709 (23.9) | 7,339 (23.8) |
| Winter | 8,522 (16.9) | 3,452 (17.6) | 5,070 (16.4) |
| Mean steps before registration  without imputation, median [IQR] | 5,233  [2,434, 8,744] | 6,280  [3,032, 10,190] | 4,663  [2,132, 7,845] |
| Mean steps before registration  with imputation, median [IQR] | 5,115  [2,428, 8,561] | 6,159  [2,993, 10,065] | 4,556  [2,153, 7,646] |
| Mean steps after registration  without imputation, median [IQR] | 5,367  [3,429, 7,695] | 6,506  [4,350, 9,076] | 4,759  [3,057, 6,781] |
| Mean steps after registration  with imputation, median [IQR] | 5,365  [3,419, 7,685] | 6,498  [4,338, 9,060] | 4,754  [3,053, 6,776] |

SD, standard deviation; IQR, interquartile range.
