# Supplementary material for: Effects of Mobile Health Care App "Asmile" on Physical Activity of 80,689 Users in Osaka Prefecture, Japan: Longitudinal Observational Study
Source: J Med Internet Res. 2025 May 21;27:e65943. doi: 10.2196/65943 (PMC12138302; doi:10.2196/65943)
Supplement: Multimedia Appendix 5 [file jmir_v27i1e65943_app5.docx]

Multimedia Appendix 5

Results of all estimates of the cumulative effects of increased step count at 56, 84, and 112 days of follow-up.

|  | 56 days | | 84 days | | 112 days | |
| --- | --- | --- | --- | --- | --- | --- |
|  | Mean | 95%CI | Mean | 95%CI | Mean | 95%CI |
| All | 18,929 | 17,947–19,911 | 25,219 | 23,326–27,112 | 30,818 | 27,809–33,826 |
| Sex |  |  |  |  |  |  |
| Men | 19,277 | 17,458–21,095 | 25,926 | 22,462–29,389 | 33,262 | 27,751–38,772 |
| Women | 18,710 | 17,593–19,827 | 24,770 | 22,592–26,947 | 29,261 | 25,806–32,716 |
| Age |  |  |  |  |  |  |
| 20–29 | 22,845 | 18,365–27,325 | 26,838 | 17,735–35,941 | 35,706 | 20,765–50,647 |
| 30–39 | 19,208 | 16,112–22,303 | 23,972 | 17,734–30,209 | 28,197 | 18,071–38,323 |
| 40–49 | 20,813 | 18,824–22,802 | 28,967 | 25,063–32,870 | 35,478 | 29,055–41,902 |
| 50–59 | 20,487 | 18,636–22,339 | 27,662 | 24,068–31,256 | 34,478 | 28,787–40,169 |
| 60–69 | 14,872 | 12,807–16,938 | 20,195 | 16,372–24,017 | 23,971 | 18,031–29,912 |
| 70–79 | 17,489 | 14,516–20,463 | 23,497 | 17,935–29,059 | 29,279 | 20,818–37,741 |
| Fiscal Year |  |  |  |  |  |  |
| 2020 | 29,511 | 28,035–30,987 | 39,688 | 36,806–42,569 | 46,917 | 42,245–51,589 |
| 2021 | 14,602 | 12,064–17,140 | 20,008 | 15,268–24,749 | 27,387 | 20,071–34,704 |
| 2022 | 10,968 | 9,033–12,903 | 14,285 | 10,580–17,989 | 17,781 | 12,109–23,452 |
| 2023 | 11,318 | 8,910–13,726 | 17,214 | 12,550–21,878 | 23,436 | 15,750–31,122 |
| Season |  |  |  |  |  |  |
| Spring | 28,553 | 27,223–29,884 | 37,805 | 35,213–40,397 | 40,077 | 35,948–44,206 |
| Summer | 3,304 | 1,034–5,574 | 9,604 | 5,240–13,968 | 24,612 | 17,715–31,509 |
| Fall | 13,556 | 10,972–16,140 | 12,108 | 7,128–17,087 | 11,934 | 4,315–19,552 |
| Winter | 15,601 | 13,314–17,888 | 27,715 | 23,659–31,772 | 42,381 | 35,752–49,011 |
| Baseline Steps |  |  |  |  |  |  |
| –1999 | 50,635 | 48,444–52,825 | 82,407 | 78,201–86,613 | 117,457 | 110,984–123,931 |
| 2000–3999 | 41,773 | 40,318–43,227 | 65,219 | 62,415–68,024 | 89,147 | 84,591–93,704 |
| 4000–5999 | 25,338 | 23,616–27,059 | 36,414 | 33,099–39,729 | 45,654 | 40,407–50,901 |
| 6000–7999 | 6,312 | 4,020–8,604 | 4,978 | 549–9,408 | 3,802 | -3,078–10,682 |
| 8000–9999 | -7,039 | -10,612–-3,466 | -14,583 | -21,423–-7,743 | -21,904 | -32,689–-11,120 |
| 10000–11999 | -18,802 | -24,207–-13,397 | -37,665 | -47,888–-27,442 | -55,331 | -71,200–-39,462 |
| 12000+ | -47,454 | -54,994–-39,914 | -83,095 | -96,378–-69,812 | -119,588 | -140,021–-99,154 |

95%CI, 95% confidence interval.
